# Effect of educational status on performance of older adults in digital cognitive tasks: A systematic review

**DOI:** 10.1590/1980-57642016dn11-020003

**Published:** 2017

**Authors:** Lucas Pelegrini Nogueira de Carvalho, Diana Quirino Monteiro, Fabiana de Souza Orlandi, Marisa Silvana Zazzetta, Sofia Cristina Iost Pavarini

**Affiliations:** 1 Mestrando do Programa de Pós-graduação em Enfermagem - Universidade Federal de São Carlos, SP, Brazil; 2 Professor Adjunto do Curso de Graduação em Gerontologia - Universidade Federal de São Carlos, SP, Brazil; 3 Professora Titular do Curso de Graduação em Gerontologia - Universidade Federal de São Carlos, SP, Brazil

**Keywords:** older adults, cognition, years of education, digital tests, idosos, cognição, escolaridade, testes digitais

## Abstract

As people age, cognitive abilities may decline resulting in serious disabilities. Neuropsychological instruments can provide information on the cognitive state of older adults. Researchers worldwide have been using digital cognitive tests to assess cognitive domains.

**Objective:**

To determine whether educational status affects the performance of older adults on digital cognitive tasks.

**Methods:**

A systematic review of articles in English, Portuguese, or Spanish published in the last 5 years was conducted. The databases searched were SCOPUS, PubMed, Lilacs, Scielo and PsychInfo. The PRISMA method was used.

**Results:**

A total of 7,089 articles were initially retrieved. After search and exclusion with justification, seven articles were selected for further review.

**Conclusion:**

The findings revealed that researchers using digital tasks generally employed paper-based tests to compare results. Also, no association between years of education and test performance was found. Finally, a dearth of studies using digital tests published by Brazilian researchers was evident.

## Abstract (pt)

A medida que as pessoas envelhecem, suas habilidades cognitivas podem diminuir, resultando em sérios problemas. Instrumentos neuropsicológicos podem fornecer informações sobre o estado cognitivo dos idosos. Pesquisadores de todo o mundo têm usado testes cognitivos digitais para avaliação da cognição.

**Objetivo:**

Identificar se a escolaridade interfere no desempenho de idosos em tarefas cognitivas digitais.

**Métodos:**

Foi realizada uma revisão sistemática dos últimos cinco anos. Foram escolhidos os idiomas português, inglês e espanhol. As buscas foram realizadas nas bases de dados SCOPUS, PubMed, LILACS, SciELO e PsycINFO. Foi utilizado o método PRISMA.

**Resultados:**

Foram encontrados 7.089 artigos, inicialmente. Após as buscas e exclusões de artigos com justificativa, foram selecionados sete artigos para descrição.

**Conclusão:**

Foi possível perceber que, ao utilizar um teste digital, o (a) pesquisador (a) também utiliza testes de papel convencionais para comparação dos resultados. Além disso, não encontrou-se associações entre os testes cognitivos digitais e a escolaridade dos participantes. Por fim, foi possível observar que pesquisadores brasileiros não tem publicado, com frequência, estudos com testes digitais como método de estudo nas bases de dados e período indicados nesta revisão.

## INTRODUCTION

Recently, the aging process has been the focus of much attention. The topic is prevalent in the media, organizations, politics, and disseminated among societies. This is due to the demographic transition, a worldwide phenomenon. Aging is a multifactorial, progressive and dynamic process occurring in the population, associated with some sociodemographic factors that warrant attention.^1^

Some of the biological events involved in the aging process are well-described in the literature.^2-4^ These events include telomeres shortening, decreased homeostasis, as well as decline in most of the organism's systems (i.e. nervous system, cardiovascular system). However, there are other psychosocial aspects that must also be considered. The trajectories, experiences, culture, and ethnicity of individuals, for example, determine the manner in which they age. This gives rise to a clear heterogeneity among the elderly population.

In Brazil, the process of population aging is intense and marked.^2,5-7^ where a decrease in births coupled with an increase in life expectancy are the main factors driving this process.^5,8^ According to the 2010 national census (IBGE, 2011), 10.8% of the Brazilian population is aged 60 years or over.^9^ Thus, the intensity of the aging process in Brazil is startling compared with developed countries, where the process had a slow and gradual pattern. Küchemann (2012) showed that, between 1980 and 2005, the elderly population in Brazil rose by 126.3%, while the total population increased at less than half this rate.^7^ Also, according to Doll et al. (2015), by the year 2030, there will be more older adults than children aged less than 14 years old. Moreover, by the year 2060, projections show that more than one third of the population will comprise elderly citizens. Therefore, the older population is set to increase substantially over the short term. Hence, both society and families must be prepared for these demographic changes.^7^

Besides these population changes, another much discussed topic regarding the aging process is cognition. It is known that the aging brain decreases in size and weight.^4^ This is due to cell loss, which leads to gyri shrinkage, as well as sulci and ventricle enlargement.^9^ This age-related cell loss is not uniform throughout the brain, affecting mostly the frontal lobe and hippocampus.^4,10^ According to recent research, normal cognitive decline caused by these brain alterations can occur with aging.^11^ Episodic and working memory, attention, perception, and executive functions are the most commonly affected cognitive domains.^11,12^ However, as Bjourklund (2015) suggests, older adults also exhibit improvements in cognition.^3^ She highlighted brain plasticity and faster decision-making as benefits of the aged brain. Similarly, Ober (2010) holds that memory will not decline to a point where activities of daily living (ADL) are impaired.^13^ Indeed, as stated above, in normal aging only episodic memory and working memory actually decline, while some types of memory remain unimpaired. In fact, studies show that semantic memory tends to improve with age.^3,13^

Because older age is a risk factor for developing mild cognitive impairment (MCI) and dementias (due to Alzheimer's Disease, for example), it is necessary to track cognitive alterations in the aging brain to prevent worse decline. Cognitive screening is an important tool for identifying cognitive profiles, allowing interventions to be planned when necessary. However, Reppold et al. (2015) outlined some shortcomings of psychometric instruments. According to the authors, the most used instruments are designed to assess memory and language.^14^

Also, it has been shown in the literature that a subject's educational status may affect the results of assessments. This is known as educational bias. Zimmermann et al. (2015) performed a study on the effects of age and years of education on older adults' performance for executive functions.^15^ In the study, the authors used the Wisconsin Card Sorting test, Digit Span test, and the Stroop task. The results suggested a significant difference for educational status, yet smaller, non-statistically significant disparities for age and gender. Similarly, Steibel et al. (2016) investigated the influence of age and years of education on performance in the Rivermead Behavioral Memory Test (RBMT). They found a negative correlation between age and test performance, and a positive correlation between educational status and performance.^16^

There seems to be a growing need for sensitive and reliable instruments to evaluate cognition in the elderly population with different educational status. This is especially true because of the longevity and high prevalence of cognitive impairment among older adults. Researchers from developed countries have used a technological approach to test cognitive domains. Computers, tablets, and video games have been used as methodological tools, and sometimes even as substitutes for traditional paper-based neuropsychological tests. In Brazil, however, this practice has not yet been widely adopted by researchers. Most of the instruments employed are not in a digital format, where the use of technology appears to be a promising way forward for the implementation of instruments among the elderly population with different educational backgrounds.

Therefore, the aim of this systematic review was to determine whether educational status affects the performance of older adults on digital cognitive tasks.

## METHODS

A systematic review of the literature was conducted for studies from the past 5 years based on the search topic of cognitive evaluation using digital tasks in older adults. The search was performed in October 2016 on the following databases: SCOPUS, PubMed, Lilacs, Scielo and PsychInfo. The descriptors were obtained from DeCS and MeSH, and were "escolaridade", "cognição", "idoso" e "testes neuropsicológicos".

**Figure 1:**
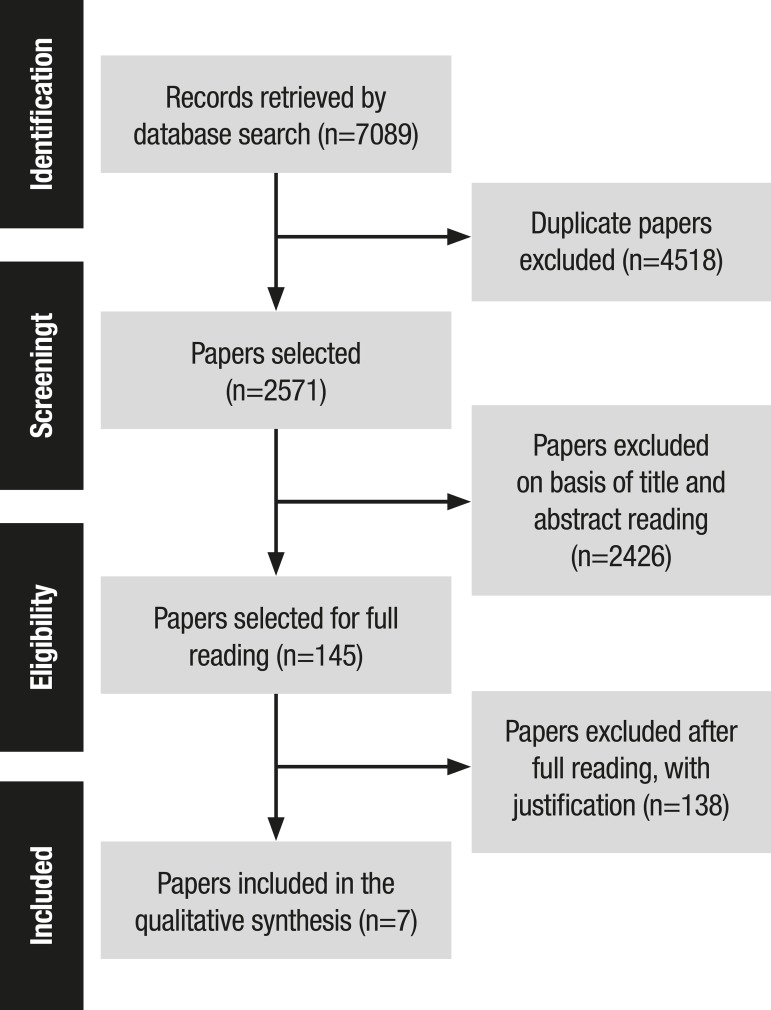
Illustrative Summary of Paper Selection process for the Systematic Review. PRISMA Method.

The search strategies were combined based on the above-mentioned descriptors. The Boolean operator "AND" was used to set the combinations, which were: "Ancianos AND Escolaridad AND Cognición"; "Idosos AND Escolaridade AND Cognição"; "Aged AND Educational Status AND Cognition"; "Aged AND Educational Status AND Neuropsychological Tests"; "Idosos AND Escolaridade AND Testes Neuropsicológicos"; "Ancianos AND Escolaridad AND Prubas Neuropsicológicas"; "Older Adults AND Years of Education AND Neuropsychological tests".

In order to make the search more precise, the following filters were used: papers published between 2012 and 2016; papers written in Portuguese, English, and Spanish. On SCOPUS, the search was carried out using title, abstract, and key-words; also, the document type was paper. On PubMed, papers were requested, and the search was by title and abstract. On Scielo, papers were searched for by title. Finally, on Lilacs and PsycInfo the studies were searched for by all fields.

The inclusion criteria were the following: publications from the past 5 years; in English, Spanish, or Portuguese; cognitive assessment performed using a digital task; availability (open access); and sample containing older adults (aged 60 years or older). The excluded papers were those whose fields essentially involved genetics, or for which the cognitive assessment was based on paper-based neuropsychological tests only, or whose subjects were not older adults, or with studies using pharmacological treatment.

For the selection process, the Preferred Reporting Items for Systematic Review and Meta-Analysis (PRISMA) system was used. Its purpose is to help authors improve reporting of previously published systematic reviews. Moreover, data analysis and extraction were done using an adapted form of the instrument proposed by Ursi (2005).

## RESULTS

The search on the databases led to the retrieval of 7,089 papers. Of this total, 4,518, both inter and intra database, were excluded for duplication. A further 2,426 papers were rejected after reading of the title/abstract because they did not meet the inclusion criteria. Subsequently, 145 studies were selected for full reading, 138 of which were excluded. Finally, seven studies were selected for inclusion in this review.

Out of the selected papers, only one was conducted in Brazil, more specifically, in the state of Sao Paulo.^17^ Regarding age of the samples, the majority had a mean population age of 70 years or older. Also, the studies had some limitations, such as missing evaluations and death.^17-20^

Most of the studies used the Mini-Mental State Examination (MMSE) as exclusion criteria. In addition, other tests, such as the MoCA (Montreal Cognitive Test) and the Geriatric Depression Scale (GDS), were used to complement the digital tasks.^17,20-^22^,24^ With respect to educational background, most of the elderly had more than 7 years of education.^17,18,20,23,24^ Another characteristic was that most of the studies included participants with some cognitive impairment.^18,23,24^

Table 1 shows the information obtained from the papers selected for the systematic review.

**Table 1:** Studies using digital cognitive tests in older adults.

Study	Place	Demographics	Cognition measurement	Years of education	Main findings
Zorluoglu et al. (2015)	Turkey	N = 23Age: 81.78	• MoCA + Computer-Based Neuropsychological tests	Control = 13.66Mean = 13.71	MoCA mean = 13.57 MoCA control = 24.55 MCS mean = 19.92 MCS control = 26.88
Genon et al. (2013)	Belgium	N = 32	• Controlled Episodic Retrieval (CER)+ Recollection digital tests	AD+ = 11.9 (3.5)AD- = 13.2 (4)	AD patients had deficient functional connectivity during associative CER, suggesting that the residual recollection function in these patients might be impoverished by the lack of some recollection-related aspects such as autonoetic quality, episodic details and verification.
Brambati et al. (2012)	Canada	N = 13Age: 72.7	• Semantic priming• Repetition priming	14.7	The results showed a defective priming effect in Mild Cognitive Impairment in the semantic but not the repetition priming condition.
Oliveira et al. (2014)	Sao Paulo, Brazil	N = 20Age: 77.5	• Sociodemographic questionnaire • MMSE • GDS +Trail Making A • Trail Making B • Spatial Recognition • Go/No-Go • Pattern Recognition • Memory Span • Reverse Memory Span	7.75	There was a learning effect when the results were compared only for the Trail Making Test A. No significant performance changes were evident on the other tests.
Richards et al. (2014)	England, Scotland, and Wales	N = 1668Age: 60-64	• 15-item word learning task (devised by the NSHD) + Visual Searching Task	-	Affective symptoms were more clearly associated with self-reported memory problems in late midlife than with objectively measured cognitive performance.
Nystrom et al. (2015)	Sweden	N = 70	• Stepwise comparative status analysis • To assess basic cognitive symptoms I-Flex • MMSE • Clinical dementia rating	7-22	When adjusted for age and education, MCI-vas performed significantly worse than MCI-nov patients on memory, language, and executive tests.
Xie et al. (2015)	United States	N = 57	• MMSE • Rey Auditory Verbal Learning Test • Rey-Osterrieth Complex Figure Trail Making Test Parts A and B, Letter-Number Sequencing • Frontal Assessment Battery • Stroop Color-Word Test	-	The Automated Neuropsychological Assessment Metrics was more effective than the MMSE for detecting CI, but further research is needed to develop a more optimal cognitive screen for routine use in heart failure patients

MoCA: Montreal Cognitive Assessment; MCS: Mobile Cognitive Screening; MMSE: Mini Mental State Examination; GDS: Geriatric Depression Scale; ADAS-Cog: Global Cognitive function measured by the Chinese version of the Alzheimer's Disease Assessment Scale - Cognitive Subscale.

Zorlouglu et al. (2015) developed a Mobile Screening Test (MST), and performed a study comparing performance on this task and the MoCA between two groups: dementia group and control group (healthy older adults). According to the authors' findings, there were no differences in performance between the two tests and the MST correlated with the MoCA (r^2^= 0.57/p<0.01).^20^ Moreover, the MST results were able to differentiate individuals with dementia from controls. The authors did not mention any educational bias on the test. However, they noted that arithmetic tasks were thought to be easy due to participants' high educational status.

In 2013, Genon et al. conducted a study to observe the brain regions related to recollection in Alzheimer's disease (AD) patients and healthy controls. Although the digital task had words, participants showed no issues on this paradigm. Accordingly, it is important to note that this was a Belgian study involving participants whose mean education was 11.9 (3.5) and 13.2 (4.0) years for AD and control groups, respectively.^24^

Brambati et al. (2012) performed a study evaluating the integrity of the semantic memory system in older adults with amnesic MCI (aMCI). In order to gather this information, they designed the study with two groups (aMCI and control) and also used a digital priming test under two conditions: semantic and repetition. Following a similar pattern to the studies mentioned above, participants had a high educational level. Mean years of education for the aMCI group was 14.8 (3.9) and for the control group was 12.2 (2.6) years.^18^ The authors found no associations or correlations between test performance and years of education.

An interesting study carried out by Brazilian researchers evaluated the learning effect of digital tests among the elderly.^17^ To this end, Oliveira et al. (2013) used digital tests developed by Sternberg et al. (2013). According to their results, age and years of education had no impact on subjects' performance. In summary, the authors concluded that only the Trail-making test A showed a learning effect, whereas the others did not.^17^

Another study conducted by Richards et al. (2014) tested prospective associations between cognitive function in late middle age and life-course affective symptoms. This group found no correlations or associations between years of education and digital test performance. Likewise, Nyströn et al. (2015) also failed to find such correlations.

Finally, Xie et al. (2015) sought to determine the evaluation ability of a digital neurological battery (ANAM) among heart failure patients. They tested 57 patients aged between 45 and 90 years. The authors found that ANAM accuracy and efficiency was not associated with educational status (p>0.05), but was weakly associated with age (r= -0.36/ p<0.01; r=-0.27/ p<0.05).^22^

## DISCUSSION

This systematic review is the first study that analyzes older adults' performance on digital cognitive tests. The main results from this study show that there is little research using computer-based tasks evaluating cognition among the elderly population in Brazil. Although educational status and cognition is a well-explored field, there is still a need for further studies comparing subjects' performance on digital tasks and their educational backgrounds.

The data reveals the importance of educational development, even in elderly individuals, because mental capacity reflects aspects such as education, social status, and environment circumstances.^24,25^ Educational background has been found to play a major role in elders' intellectual ability.^24-26^

Also, the aging process may lead to cognitive impairments, commonly observed as difficulties remembering recent events, doing math, and attentional problems.^27-29^ According to Souza et al. (2013), education influences intelligence more than aging. Thus, education is a factor determining performance of cognitive abilities that warrants attention, especially among the elderly population.^30^ However, it is noteworthy that, besides educational background, age is also an important determinant of cognitive decline.^29,31-33^ However, cognitive decline is clearly more prevalent among older subjects, especially those aged 65 years or older.^34,35^

Cognitive function can be measured by various means, where the MMSE is the most used instrument because it can track cognitive dysfunctions. Furthermore, the MMSE is able to detect subtle changes and also investigates the prevalence and incidence of the dementia process.^29,36^

Neuropsychological instruments require a higher educational level, and formal education is the variable that has the greatest impact on cognition.^35,37^ Both the evaluation and treatment of cognitive domains are growing fields and, due to technological improvements, this topic has attracted interest from researchers worldwide. According to Negut (2014), traditional paper-and-pencil evaluations have been criticized due to their lack of precision for activities of daily living. Consequently, the number of cognitive assessments and evaluations employing digital tests has increased.^38^ The aim of this type of evaluation is to promote more efficient, practical and inexpensive neurocognitive assessment.^39,40^

Despite the known benefits of traditional paper-based tests, digital cognitive tasks seem to be a valid alternative for assessing cognitive performance, not only for their ease of use and data storage, but also because they can improve patient treatment and motivation.^40,41^

To conclude, the main purpose of this review was to determine whether there is an educational bias or effect of educational background on older adults' performance in digital tests. In all studies reviewed, no association between participant performance and years of education was found. Furthermore, despite the scant publications on this topic, evidence from this study suggests the inexistence of educational bias in digital cognitive tests.

The findings showed that researchers using digital tasks tend to also use paper-based test to compare results. This is probably due to the fact that there is not yet a gold standard for digital tasks. Thus, further research should be done in order to provide researchers with this information.

Finally, it is notable that only one of the selected papers was Brazilian. Technology associated with measuring tools appears to be a promising way forward in the cognitive field. Indeed, researchers from developed countries, such as the United States, Canada, and United Kingdom, have made increasing use of digital tests since the 1970s. Hence, greater use of digital tasks in Brazil is needed in order to accompany international trends in research.
